# Progress in Surgery

**Published:** 1900-06-02

**Authors:** 


### Progress in Surgery.

### PROGRESS IN OBSTETRICS.

**Eclampsia.**—Dr. Davis,[1] after pointing out the inadequacy of previous theories as to the causation of this disease, states that the weight of evidence is distinctly in favour of the belief that a profound toxæmia originating within the bodies of mother and fœtus is the cause of eclampsia. The urine of eclamptics injected into animals is but feebly toxic. But this is because the toxins of eclampsia while absent from the urine are retained in the blood serum, and in the organs of the body. Abuladse[2] insists that eclampsia is a primary disease of the kidneys due solely to auto-infection of the patient by the accumulation of waste products in the maternal and fœtal blood. Levniovitsch[3] believes there is a definite germ in the blood in eclampsia. He found it constantly in 44 cases of puerperal convulsions. They appeared as large round and oval cocci, very automobile; and they grew best on nutritive media, consisting of placental tissue. They were sometimes found in the blood before the first fit; indeed, they were detected in several patients who had no convulsions, but suffered from headache, vomiting, and œdema. They were invariably most abundant during the fit, and in about two days after the last fit they steadily diminished in number. Subcutaneous injection of pure cultures in nongravid rabbits frequently set up tetanic spasms of short duration in different sets of muscles. Occasionally the cocci were detected in the blood of the infants of eclamptic mothers; in two infants fits occurred. Dr. Everke[4] points out that as a rule the convulsions cease as soon as the child is born, and therefore he is of opinion that eclampsia is produced by a toxic influence issuing from the fœtus, and entering the circulation of the mother through the placenta. Vorhees[5] reports an interesting case of puerperal eclampsia complicated with erythema multiforme. The convulsions ceased 36 hours after delivery, but seven days later a bullous eruption appeared on the extremities, and spread with remarkable rapidity; in a few days the trunk, face, mouth, gums, and tongue were invaded. The bullæ left ulcers when they burst. The eruption was at its height on the fourth day; the temperature 102, pulse 124. At length the disease disappeared after a few relapses, during which the bullæ appeared smaller and smaller. At the end of a month the patient was well. The erythema multiforme in a bullous form is very rare, and Vorhees cannot find a case reported associated with childbed. Hillman[6] has tabulated 40 cases of eclampsia in which Cæsarian section was performed, and thinks that if the uterus is inactive, the cervix undilated, and the mother's condition so urgent that time cannot be lost, the operation offers a fair chance of saving both mother and child. Of the 40 cases tabulated by Hillman, the maternal mortality was 51·3 per cent., and the fœtal mortality 43·9 per cent. Considering the serious nature of the conditions under which the operations were performed, the results must be regarded as encouraging, for we know that severe cases of eclampsia, in which delivery cannot be rapidly completed, nearly always terminate fatally.

**Symphysiotomy.**—Whitridge Williams[7] records an interesting case of spondylolisthesis, in which symphysiotomy was performed. The patient was a negress, aged 22, and 4 ft. 10 in. in height. Naegele pelvis was at first suspected, owing to the presence of evident asymmetrical pelvic deformity. On pelvic examination it was found that the body of the fifth lumbar vertebra had been so displaced downwards as to completely cover the anterior surface of the first sacral vertebra, its lower margin being opposite the articulation between the first and second sacral vertebræ. A spondylolisthetic pelvis was suspected, and diagnosis assured by sweeping the finger along the linea innominata, when it came in contact with the displaced last lumbar vertebra, instead of continuing on to the promontory of the sacrum. As the promontory was covered by the last lumbar vertebra, the oblique conjugate could not be measured, but the distance from the upper margin of the last lumbar vertebra to the lower margin of the symphysis pubis was 9·5 c.m. ($3\frac{3}{4}$ inches). The pelvic outlet was somewhat contracted. Symphysiotomy was performed, and the child saved. The abdominal wound partially yielded on the seventh day, when the patient died suddenly with symptoms of pulmonary embolism. The pelvis was carefully examined, and marked spondylolisthesis discovered. R. C. Buist[8] gives a short account of labour at full term in two cases in which he had previously performed symphysiotomy. The first patient was troubled during pregnancy with a good deal of pain in the lower part of the abdomen, but was delivered at term of twins without difficulty. During delivery the patient was placed in the Walcher position. One child presented by the vertex, the other by the breech, and each biparietal diameter measured 10 c.m., and the occipito-frontal circumference 35·5 c.m. The diagonal conjugate diameter of the pelvis was 9 c.m. In the second case, fibrous union only had occurred after the first operation; but the patient did well during the second pregnancy, and had not trouble beyond a little frequency of micturition. During labour the patient was placed in the Walcher position, and with the assistance of a little suprapubic pressure expulsion was effected without further difficulty. The diagonal conjugate in this case was $9\frac{1}{2}$ c.m., and the occipito-frontal circumference of the child's head 32 c.m. The puerperium was uneventful. The symphysis after delivery was a little more mobile than formerly, but it did not dilate perceptibly during labour. In both these cases there appears to have been some permanent enlargement of the pelvis as a result of the previous symphysiotomy.

**Tumours Complicating Pregnancy.** — Coe[9] reports several interesting cases of pregnancy complicated by uterine fibroids, and he points out that the rapidity of growth of a fibroid under the influence of pregnancy is in direct proportion to its more or less intimate relation to the uterus. Thus a pedunculated subperitoneal growth may remain unchanged, while an interstitial tumour undergoes marked enlargement. On the other hand, a subserous fibro-myoma situated in the lower uterine segment, or displaced and impacted in the pouch of Douglas may increase in size simply from obstruction to its circulation. Porter also lays stress on the position of the tumour, a growth in the lower segment of the uterus usually proving very serious. Gemmel[10] reports a case of hydatic cyst in the omentum which obstructed labour, and was subsequently removed by abdominal section. The patient was first seen after being in labour for 24 hours, and on examination a cystic tumour was felt low down in the pelvis; the first stage of labour was completed, and the head unable to descend into the pelvis. The tumour was the size of a cocoanut, and was, between the pains, felt to be movable; under an anæsthetic it was pushed out of the pelvis into the abdomen; after this forceps were applied to the head and the child easily delivered. The puerperium was normal. Six weeks later the abdomen was opened; both ovaries and Fallopiun tubes were found to be healthy, and the cyst, which contained jelly-like material and daughter cysts, was intimately connected with the omentum. W. S. Stone[11] describes a case of ovarian cystoma complicating pregnancy which almost completely filled the pelvis, and in which he made a posterior vaginal incision and drained the cyst. It was unilocular, but could not be completely removed on account of the pregnancy. The patient made a good recovery.

**Puerperal Insanity.**—William Hirsch,[12] writing on this subject, summarises the important points of the relation of child-bearing to mental diseases as follows: (1) A specific form of mental disease, which might be called puerperal insanity, does not exist. The different psychoses which are observed during any of the stages of gestation are the same as those we see in other patients; (2) pregnancy may, under certain circumstances, be one of the etiological factors of insanity. Its etiological importance, however, is proved by neither statistics nor clinical observation. It is, therefore, not permissible to terminate pregnancy on account of a psychosis, unless there are special indications for such intervention. (3) During parturition we sometimes meet with a passing disturbance of consciousness, the clinical features of which resemble psychical epilepsy. (4) Pyschoses which occur in connection with the act of parturition are produced (*a*) by trauma in cases of difficult labour; (*b*) by anæmia and exhaustion, after severe hæmorrhage; and (*c*) by intoxication in septic cases, or cases with local inflammation, or uræmia. (5) Lactation, as such, plays no *rôle* at all in the production of insanity. It is due to other circumstances; that, during the first few months after delivery, women on the average are more predisposed to nervous and mental diseases than under ordinary conditions.

[1] Practitioner, Dec., 1899, Dr. Davis. [2] Brit. Med. Jour., Nov. 18, 1899. Abuladse. [3] Brit. Med. Jour., Dec. 9, 1899, Levniovitsch. [4] Lancet, Dec. 23, 1899, Dr. Everke. [5] Brit. Med. Jour., Dec. 30, 1899, Vorhees. [6] Brit. Med. Jour., Dec. 9, 1899, Hillmann. [7] Brit. Med. Jour., Oct. 14, 1899, Whitridge Williams. [8] Scottish Med. and Surg. Jour., Jan., 1900, R. C. Buist. [9] New York Med. Jour., Nov. 25, 1899, Coe. [10] Brit. Med. Jour., Dec. 9, 1899, Gemmel. [11] Med. Rec., Nov. 11, 1899, W. S. Stone, [12] Med. Rec., Jan. 6, 1900, William Hirsch.

